# Glove and instrument handling in small animal oncological surgeries: a survey

**DOI:** 10.1111/jsap.13852

**Published:** 2025-03-18

**Authors:** E. Orjefelt, J. R. D. MacKay, K. L. Bowlt Blacklock

**Affiliations:** ^1^ Royal (Dick) School of Veterinary Studies Edinburgh UK

## Abstract

**Objectives:**

To evaluate veterinary surgeons' awareness of the potential for surgical gloves and instruments to act as vectors for tumour seeding in small animal oncological surgery and to assess the use of specific protocols to mitigate this risk.

**Materials and Methods:**

A 21‐question survey was developed and distributed to small animal veterinary surgeons, focusing on practices related to glove and instrument handling during oncological surgeries. The survey targeted veterinary surgeons who regularly performed oncological procedures, and was analysed using descriptive statistics and Pearson's chi‐square analysis.

**Results:**

A total of 194 veterinary surgeons participated. Most respondents (89%) reported changing gloves and instruments during oncological surgeries to avoid tumour seeding. Surgeons with advanced qualifications and those working in referral hospitals were more likely to implement these practices. Additionally, surgeons with a higher oncological caseload were more likely to follow protocols for wound protection. The majority (74%) of respondents believed that there was a risk of neoplastic cells on gloves or instruments, and 98% expressed a strong interest in evidence‐based guidelines.

**Clinical Significance:**

This study highlights a significant awareness among veterinary surgeons regarding the risk of tumour seeding through surgical gloves and instruments. There is a clear willingness among practitioners to adopt new guidelines and improve practices based on emerging evidence, indicating a potential shift towards more stringent protocols in small animal oncological surgeries.

## INTRODUCTION

Cancer represents one of the leading causes of death in companion dogs, accounting for 27% of all deaths in dogs in the UK and 45% of deaths in dogs 10 years or older (Adams et al., 2010; Dobson, 2013). In both veterinary and human medicine, surgery is considered the cornerstone for treating most neoplastic disease (Biller et al., 2016; Wyld et al., 2014).

Neoplastic cells are associated with a loss of cohesion and a change in their normal interaction with the underlying stroma, which contributes to increased cellular exfoliation during tumour manipulation (Janiszewska et al., 2020). Tumour cells that become dislodged during surgical procedures can migrate and colonise distant sites or adhere and grow in discontinuous tissue. In human surgery, tumour seeding in this manner has been reported following laparoscopic oncological surgeries (Paolucci, 2001), as well as following biopsy and aspiration cytology (Minaga et al., 2017; Shyamala et al., 2014). In veterinary surgery, similar experiences have also been reported (Faletti et al., 2022; Merickel et al., 2021). Surgical instruments and gloves are also proposed as vectors for viable cancer cells (Yu et al., 2014); however, there is no published evidence that such retained neoplastic cells can cause tumour seeding and recurrence.

Despite this, around half of surgeons in human healthcare change gloves or instruments, or both, as a routine component of extirpative cancer surgery (Berger‐Richardson et al., 2018). To the authors' knowledge, there has been no published examination of the practices and beliefs held by veterinary surgeons regarding glove and instrument handling and wound protection in oncological surgery.

The objective of this study was to assess beliefs among veterinary surgeons regarding the potential for gloves and instruments to act as vectors for tumour seeding. We also aimed to assess the perceived need for specific protocols for glove and instrument use during small animal oncological surgery. We hypothesised that veterinary surgeons would be aware of the potential for neoplastic cell seeding, and that those with a greater oncological caseload would be more proactive in implementing protocols to minimise the risk of seeding. We also hypothesised that veterinary surgeons would be interested in further research to help develop evidence‐based guidelines on the use of gloves and instruments during oncological surgery.

## MATERIALS AND METHODS

This study was approved by Human Research Ethical Review Committee at the University of Edinburgh (HERC_2023_004).

We developed a 21‐question survey for use by registered small animal veterinary surgeons, based on that by Berger‐Richardson et al. (2018). The survey was pilot‐tested on veterinary surgeons working at the Hospital for Small Animals at the University of Edinburgh. The survey was then made available online (JISC Online Surveys), advertised on veterinary forums and distributed via email to diplomates of the European College of Veterinary Surgeons (ECVS).

Respondents were eligible for inclusion if they were registered as a MRCVS, worked in clinical practice and regularly performed oncological surgery.

The questionnaire can be viewed in Appendix S1.

### Statistical analysis

There were four questions regarding surgeons' beliefs (‘Belief’) related to whether gloves or instruments could harbour tumour cells, and if they could, whether this might lead to wound or locoregional recurrence. These questions were rated on a 5‐point Likert‐like scale (Definitely Not, Improbable, Do Not Know, Probably and Definitely). There was a fifth Likert‐like question relating to whether participants were likely to change their practice (‘Practice Change’) if they were presented with evidence of this (Very unlikely, Unlikely, Neutral, Probably, Definitely). We were interested in differences in Belief and Practice Change across different explanatory variables, including: participant training level, years of practice, practice type, oncological caseload (%), whether or not they were an ECVS/ACVS Diplomate (Diplomate Status), their current practices regarding glove and instrument changing, and existence of current protocols in their practice. Differences in Belief and Practice Changes across these variables were analysed via Kruskal–Wallis tests, to account for the non‐parametric data. We were also interested in whether surgeon ‘Behaviours’ (having ever changed gloves or instruments during an oncological procedure to reduce the risk of tumour seeding) were related to participant training level, years of practice, practice type and oncological caseload. These data were analysed via a Pearson Chi^2^ test. All data were analysed in R (R Core Team, 2023), making use of the ‘likert’ (Bryer & Speerschneider, 2016), ‘ggstatsplot’ (Patil, 2021), ‘patchwork’ (Pedersen, 2024) and ‘tidyverse’ (Wickham et al., 2019) packages.

## RESULTS

In total, 194 small animal veterinary surgeons completed the survey. Respondent characteristics are shown in Table 1.

**Table 1:** Respondent characteristics

Characteristic		Number (%) respondents
Years in practice as a Veterinary Surgeon	<2 years	14 (7.3%)
	2 to 5 years	35 (18.1%)
	6 to 10 years	44 (22.8%)
	11 to 15 years	35 (17.6%)
	>15 years	66 (34.2%)
Type of practice	Small Animal General Practice	51 (26.6%)
	Mixed General Practice	20 (10.4%)
	Small Animal Veterinary Hospital	14 (7.3%)
	Small Animal Veterinary Hospital (Referral)	92 (47.9%)
	Small Animal University Teaching Hospital (Referral)	41 (21.4%)
Advanced training in Small Animal Surgery?	Certificate	27 (14%)
	Diplomate (ECVS or ACVS)	102 (52.8%)
	No	55 (28.5%)
	Other (including residency)	25 (13%)
Percentage of surgical caseload involving tumour resection?	<10%	43 (22.3%)
	10% to 30%	96 (49.7%)
	30% to 50%	35 (18.1%)
	50% to 70%	12 (6.2%)
	70% to 90%	5 (2.6%)
	>90%	3 (1.6%)

### Most veterinary surgeons change surgical gloves and instruments to avoid tumour seeding

First, we wanted to determine whether veterinary surgeons changed surgical gloves and instruments and their reasons for doing so. We found that 89% and 87.6% of respondents changed gloves and instruments at any point during an oncological surgery to avoid tumour seeding. The specific reasons provided for these practices are detailed in Table 2. Interestingly, in free‐text responses, some surgeons noted that they do not change gloves or instruments when performing a wide surgical resection where clear margins are expected or when the tumour is being removed *en bloc* without breaching the tumour capsule.

**Table 2:** Veterinary surgeon self‐reported protective behaviours to reduce tumour seeding during oncological surgery

Behaviour	Number (%) respondents
Change gloves	172 (89.1)
Change instruments	169 (87.6)

Decision basis for protective strategy	Gloves	Instruments
Evidence based	32 (18.6)	30 (17.8)
Anecdotal information	29 (16.9)	29 (17.2)
Clinical observation	9 (5.2)	10 (5.9)
Clinical training	100 (58.1)	98 (58)
Other	2 (1.2)	2 (1.2)

Strategies to protect the wound	Open	Laparoscopic
	67 (34.9)	18 (62.1)

### Veterinary surgeons protect the wound edges less commonly during oncological surgery

Next, we sought to determine whether veterinary surgeons were equally diligent about protecting the wound during oncological surgery. We found that respondents protected wound edges less commonly than changing gloves and instruments (Table 2). Furthermore, there was no statistical difference in how likely respondents were to protect wound edges during open surgeries compared to laparoscopic surgeries (χ^2^_Pearson_(4) = 6.95, P = 0.14).

In the free‐text responses, veterinary surgeons provided details about the methods of wound protection they employed. Reported methods for wound protection during laparoscopic surgery involved the use of protective retrieval bags, either commercially available or fashioned from the cut‐off tip of a sterile glove. Strategies utilised to protect wound edges during open surgery included separating areas on the instrument table to prevent cross‐contamination between ‘clean’ and ‘dirty’ instruments, performing surgical site lavage, minimising manipulation of the tumour and using specimen retrieval bags and/or wound retractors.

### Most veterinary surgeons believe that gloves and instruments can act as vectors for malignant cells

**FIG 1:**
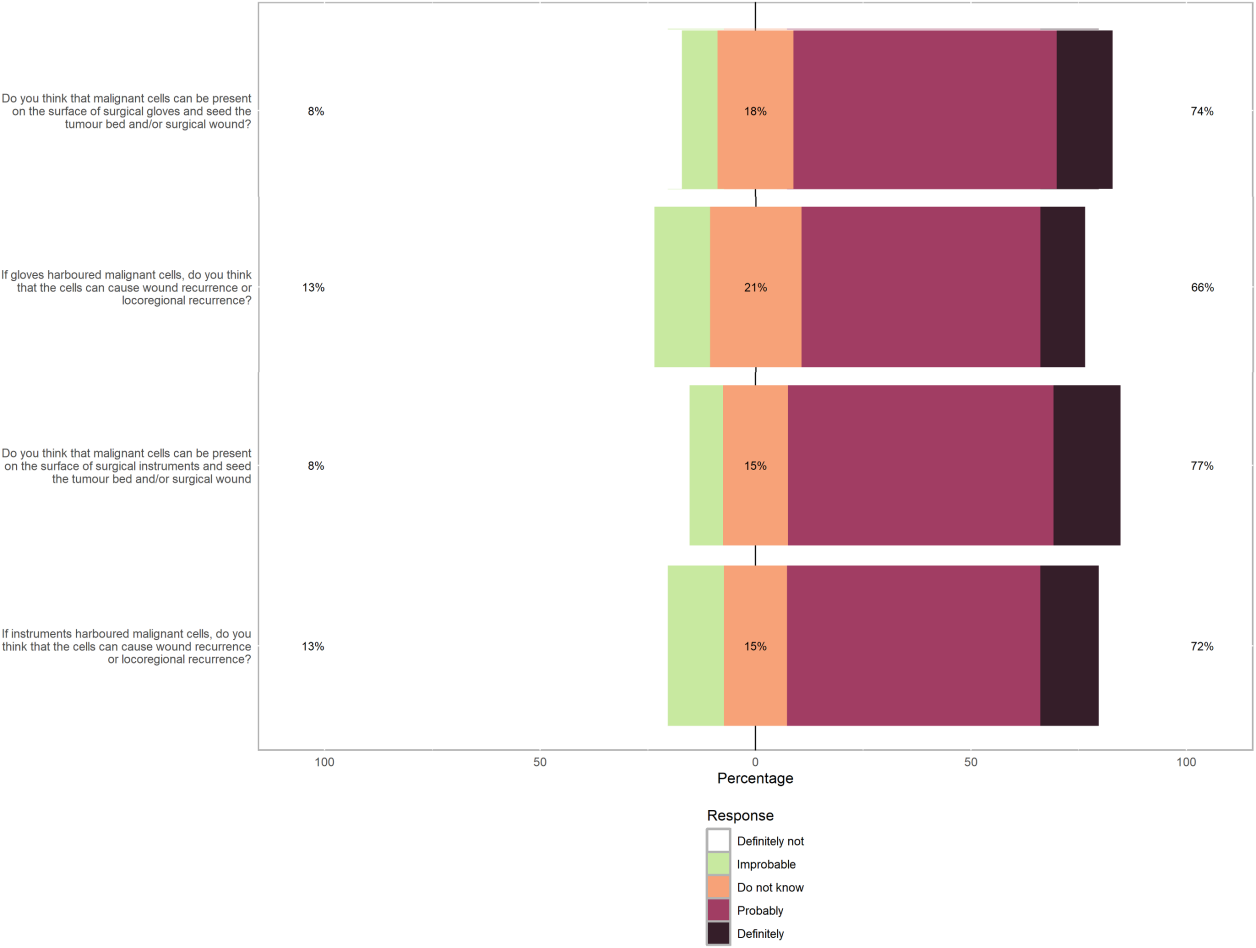
Veterinary surgeons' self‐reported beliefs about whether surgical gloves or instruments harbour malignant cells capable of leading to tumour recurrence.

We identified that most veterinary surgeons believe that neoplastic cells can be present on both surgical gloves and instruments, which can then act as potential source of cancer recurrence (Fig 1). Participants' training, Diplomate Status, practice type and oncological caseload did not influence these beliefs (all P > 0.05). We found that participants acted upon their beliefs. There were significant associations between positive beliefs that malignant cells from gloves could cause wound or locoregional recurrence and practices such as changing gloves during an oncological surgery to reduce tumour seeding (χ^2^_Kruskal Wallis_(1) = 4.54, P = 0.03) and having a protocol to protect the surgical wound from tumour seeding (χ^2^_Kruskal Wallis_(1) = 5.35, P = 0.02). Similarly, surgeon beliefs that malignant cells from instruments could cause wound or locoregional recurrence was associated with having a protocol to protect the surgical wound from tumour seeding (χ^2^_Kruskal Wallis_(1) = 4.50, P = 0.03).

### Most veterinary surgeons are amenable to changing their surgical technique when presented with evidence

**FIG 2:**
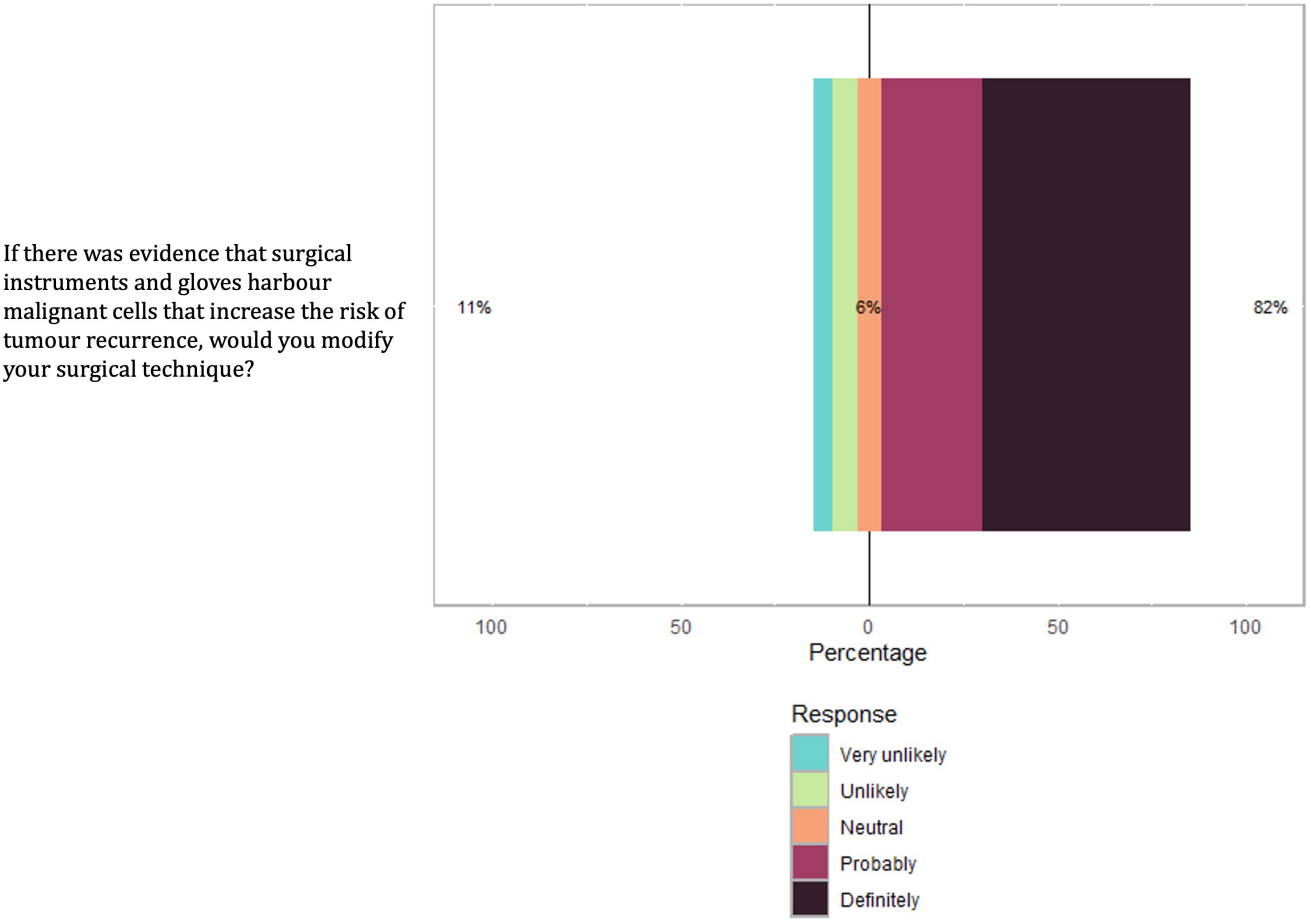
Veterinary surgeons' likelihood of modify their surgical technique during oncological surgery.

Overall, 98% of participants said that they would be interested in evidence that surgical gloves and instruments harboured malignant cells. Participants were generally inclined to modify their practice if presented with such evidence, with 82% expressing a positive attitude towards making changes. However, 5% (*n* = 9) felt very unlikely to adjust their practice even with evidence (Fig 2). Training, Diplomate Status, practice type and oncological caseload did not impact the likelihood of modifying practice in response to evidence (all P > 0.05). Those respondents who indicated they were very unlikely, unlikely or neutral in their willingness to adjust their clinical practice noted that they already changed gloves and instruments. Additionally, they mentioned that their practical decision‐making would be influenced by factors such as the type of tumour and the likelihood of achieving clean surgical margins.

### Surgeons with more experience and advanced clinical training were more likely to change gloves or instruments during oncological surgery

**FIG 3:**
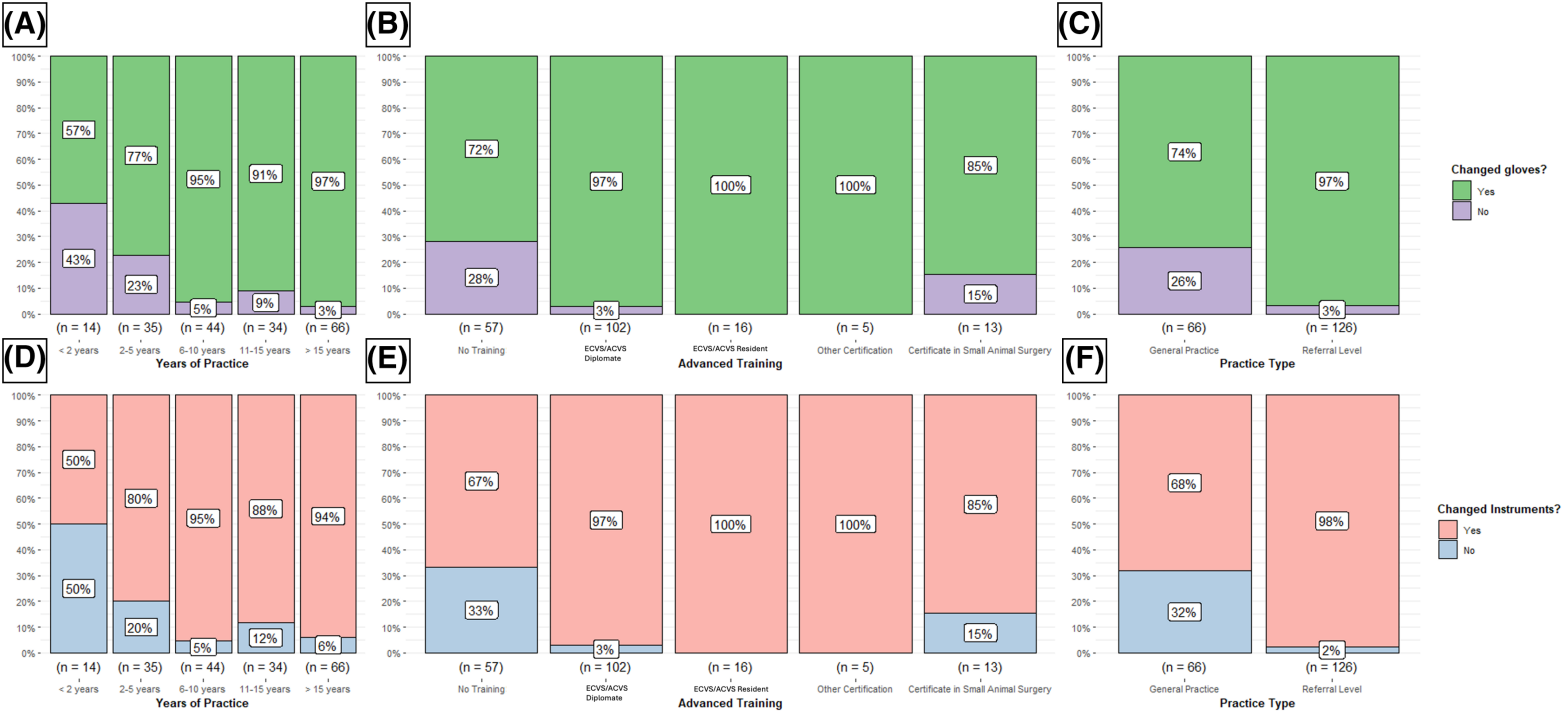
Veterinary surgeons' likelihood of modify their surgical technique during oncological surgery. Veterinary surgeons who reported changing their gloves during oncological surgery are shown by years in practice (A), advanced training (B) and type of practice (C). Similarly, those who reported changing their instruments are categorised by years in practice (D), advanced training (E) and type of practice (F).

Next, we were interested in determining which surgeon‐related variables were associated with glove or instrument changing during oncological surgery. Participants' training, their diplomate status, practice type and percentage of cases had no impact on their beliefs or likelihood to change their current protocols (all P > 0.05). However, participants who routinely changed their surgical gloves to reduce tumour seeding were more likely to have been in practice for longer (χ^2^_Pearson_(4) = 26.10, P ≤ 0.001, *V*_Cramer_ = 0.34, 95% CI [0.14, 0.47]), to have more advanced training (χ^2^_Pearson_(4) = 26.84, P ≤ 0.001 *V*_Cramer_ = 0.34, 95% CI [0.14, 0.48]) and to work at referral level (χ^2^_Pearson_(1) = 22.68, P ≤ 0.001, *V*_Cramer_ = 0.34, 95% CI [0.19, 0.48]), but were not more likely to work with a greater oncological caseload (χ^2^_Pearson_(5) = 7.90, P ≤ 0.16, *V*_Cramer_ = 0.12 95% CI [0.00, 0.48]) (Fig 3). Similarly, participants who changed their surgical instruments to reduce reseeding were more likely to have been in practice for longer (χ^2^_Pearson_(4) = 24.97, P ≤ 0.001, *V*_Cramer_ = 0.33 95% CI [0.12, 0.46]), to have more advanced training (χ^2^_Pearson_(4) = 34.39, P ≤ 0.001, *V*_Cramer_ = 0.40 95% CI [0.21, 0.53]) and to work at referral level (χ^2^_Pearson_(1) = 34.32, P ≤ 0.001, *V*_Cramer_ = 0.42 95% CI [0.27, 0.56]), but were not more likely to work with a greater oncological caseload (χ^2^_Pearson_(7.9)=, P ≤ 0.16, *V*_Cramer_ = 0.12, 95% CI [0.00, 0.25]) (Fig 3).

Importantly, surgeon variables are not independent. The number of years qualified and whether a surgeon had advanced training are related as follows: we found that, in our respondent cohort, years qualified were associated with advanced qualifications (P < 0.001). For instance, 53.8% of respondents with less than 5 years of clinical experience had no or other advanced training, compared with 7.7% holding a surgical certificate and 5.9% holding a diploma. After 15 years of clinical practice, 50% of respondents held a diploma, 38.5% held a certificate, and 12.8% had no or other advanced training. There was no difference in advanced training between 6 and 15 years of practice. Similarly, postgraduate training was statistically related to the type of practice (P < 0.001). For example, 67.6% of diplomates worked in a small animal veterinary hospital (referral) compared with 23.1% of certificate holders and 17.9% with no or other advanced qualification. In contrast, only 2.9% of diplomates worked in a small animal general practice, compared with 53.8% of certificate holders and 46.2% with no or other advanced qualification.

## DISCUSSION

Our primary aim was to evaluate the beliefs and practices of veterinary surgeons regarding the potential for surgical gloves and instruments to act as vectors for tumour seeding during small animal oncological surgery. Our findings indicate that most respondents are cognisant of a potential risk of neoplastic cell transmission via surgical tools, with 89% and 87.6% reporting the routine changing of gloves and instruments, respectively. Moreover, we highlight the influence of advanced qualifications and practice settings on these preventive measures, as surgeons with specialised training and those in referral hospitals were more likely to implement such protocols. Importantly, our results underscore the veterinary community's readiness to adopt evidence‐based practices to minimise the risk of tumour recurrence.

Our study was based on the work of Berger‐Richardson et al. (2018), who surveyed the practices and beliefs of general surgeons in Ontario. In this study, only 52% and 40% of surgeons reported that they changed gloves and instruments, respectively, with the intent of decreasing the risk of tumour seeding. This is considerably lower than the percentages observed in our study. Such a marked discrepancy may reflect differences in the perceived risks associated with tumour seeding in veterinary versus human medicine, variations in training or clinical guidelines or a higher level of awareness and caution among veterinary surgeons regarding the potential for tumour seeding during oncological surgeries. We identified similarities between our respondents and the human surgeons surveyed by Berger‐Richardson et al. (2018), including the finding that both human and veterinary surgeons with advanced training were more likely to change instruments. This may be a result of surgeons with advanced training possessing increased awareness of the potential for tumour seeding, and/or having more resources at their disposal, making a change of instruments and gloves more feasible and financially viable.

It is particularly interesting that, despite a high number of veterinary surgeons believing in the potential for tumour seeding via surgical gloves and instruments, only 34.9% and 62.1% reported using protocols to protect the wound edges during open or laparoscopic surgery, respectively. This also mirrors findings in human medicine: when asked if measures other than glove and instrument changing were used specifically to protect surgical wounds from tumour seeding during cancer surgery, 31% and 73% of surgeons reported taking additional precautions for open and laparoscopic procedures, respectively (Berger‐Richardson et al., 2018). The reason for this disparity between open and laparoscopic surgery is unclear, but is likely due to the wave of reports which noted port‐site metastasis after laparoscopic removal of incidental gallbladder cancer that commenced in human health care in the 1990s, resulting in calls for the routine use of specimen retrieval bags (Berger‐Richardson et al., 2017; Kemp et al., 2008; Paolucci, 2001). Similar reports or publicity following open surgery are lacking.

The current lack of evidence on whether gloves and instruments harbour malignant cells influences surgeons' attitudes towards modifying their surgical techniques. Recent research in human oncological surgery has demonstrated that neoplastic cells can be detected in up to 26% of surgical glove washings and 34% of instrument washings across various cancers, including head and neck squamous cell carcinoma, gastrointestinal adenocarcinoma, mesothelioma and soft tissue sarcoma (Berger‐Richardson, n.d.; Kuhar et al., 2021). These neoplastic cells can survive on stainless steel and latex gloves for 10 to 20 minutes, potentially serving as vectors for tumour seeding (Berger‐Richardson et al., 2017). Although there is no conclusive evidence that such cells result in tumour recurrence in the wound bed, a cautious approach is advisable. Given the minimal cost associated with avoiding the reuse of ablative instruments and the relative availability of surgical instruments, current recommendations in human surgical healthcare advocate for changing gloves and instruments during oncological surgery, although this should be balanced against sustainable practice and minimising waste (Meyer et al., 2022). Interestingly, this precaution may only be necessary for more malignant tumours or when the tumour capsule is breached, as the risk of harbouring malignant cells on gloves and instruments appears minimal in margin‐negative, curative wide local excisions of extremity sarcomas (Berger‐Richardson et al., 2017). However, similar studies in veterinary surgery are lacking, and no studies in human or veterinary health care involve long‐term follow‐up surveillance of surgical wounds for assessment of cancer recurrence.

Both human and veterinary surgeons are amenable to change when evidence is forthcoming: 94% and 82.5% of human and veterinary surgeons expressed willingness to change their behaviours regarding glove and instrument handling in cancer surgery if presented with evidence of potential benefit (Berger‐Richardson et al., 2018). The reason for the lower positive response in veterinary surgeons may be because most of our respondents are already changing gloves and instruments, suggesting a more proactive approach in veterinary surgery towards minimising the risk of tumour seeding.

## LIMITATIONS

While this study provides valuable insights into the awareness and practices of veterinary surgeons regarding the potential for tumour seeding via surgical gloves and instruments, it is not without limitations. Firstly, our survey relied on self‐reported data, which may be subject to response bias, as respondents might have overreported practices perceived as desirable or underreported those viewed less favourably. Additionally, the sample size is not fully representative of the entire population of veterinary surgeons, especially those who may not regularly engage in online forums or email lists through which the survey was distributed. Finally, while our study highlights the interest in evidence‐based guidelines, it does not directly assess the impact of current practices on clinical outcomes, such as recurrence rates, limiting the ability to correlate reported practices with clinical efficacy. Future research which measures actual contamination of gloves and instruments with cancer cells, is essential to provide a more comprehensive understanding of the potential risks and necessary protocols in oncological surgery.

In conclusion, this study reveals a high level of awareness among veterinary surgeons about the potential for tumour seeding via surgical gloves and instruments in small animal oncological surgery. The findings suggest that advanced training and a high oncological caseload are significant factors influencing the adoption of protocols to mitigate this risk. Despite the existing variability in practices, there is a strong interest among veterinarians in adopting evidence‐based guidelines to standardise procedures and reduce the risk of tumour recurrence. This underscores the importance of developing and disseminating clear, evidence‐based protocols to enhance surgical outcomes in veterinary oncology. Future research should focus on establishing these guidelines and further investigating the effectiveness of specific protocols in preventing tumour seeding, thus contributing to the overall improvement of cancer care in veterinary practice.

### Author contributions

**K. L. Bowlt Blacklock:** Conceptualization (equal); data curation (equal); formal analysis (equal); methodology (equal); project administration (equal); resources (equal); software (equal); supervision (equal); validation (equal); visualization (equal); writing – original draft (equal); writing – review and editing (equal). **J. R. D. MacKay:** Data curation (equal); formal analysis (equal); software (equal); validation (equal); visualization (equal); writing – original draft (equal); writing – review and editing (equal). **E. Orjefelt:** Data curation (lead); investigation (equal); writing – original draft (equal); writing – review and editing (equal).

### Conflict of interest

None of the authors of this article has a financial or personal relationship with other people or organisations that could inappropriately influence or bias the content of the paper.

## Supporting information


**Appendix S1.** Questionnaire to determine veterinary surgeons' practices relating to glove and instrument handling during oncological surgeries.

## Data Availability

Data is available upon request from the corresponding author.
