# Supplementary figures and images for: Glove and instrument handling in small animal oncological surgeries: a survey
Source: J Small Anim Pract. 2025 Mar 18;66(7):477–83. doi: 10.1111/jsap.13852 (PMC12232354; doi:10.1111/jsap.13852)

Appendix 1.


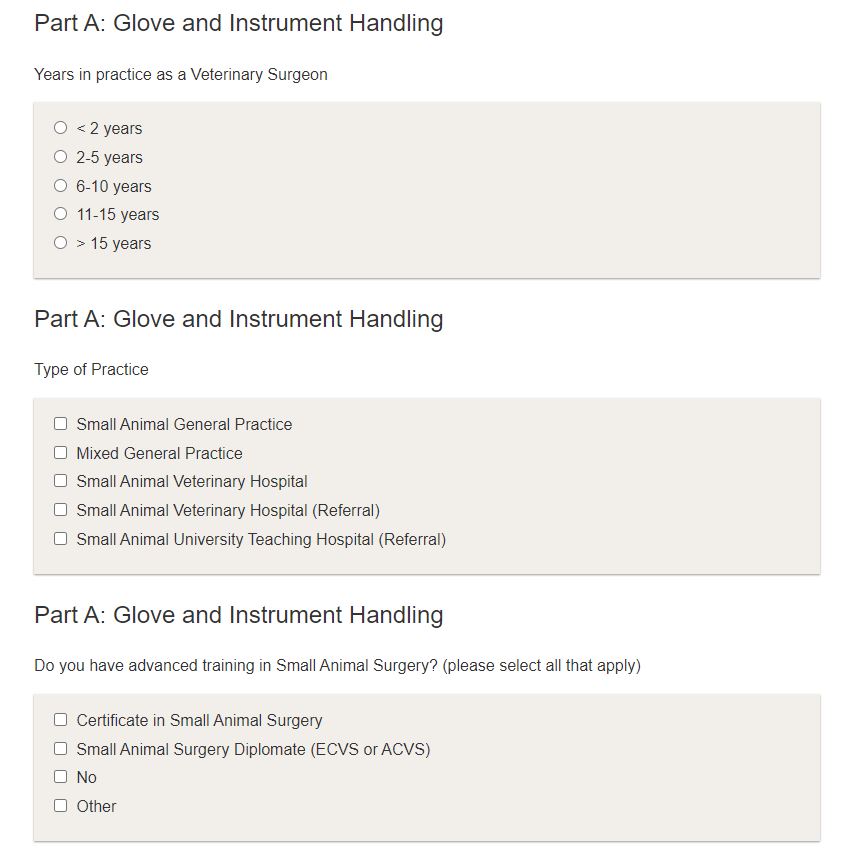


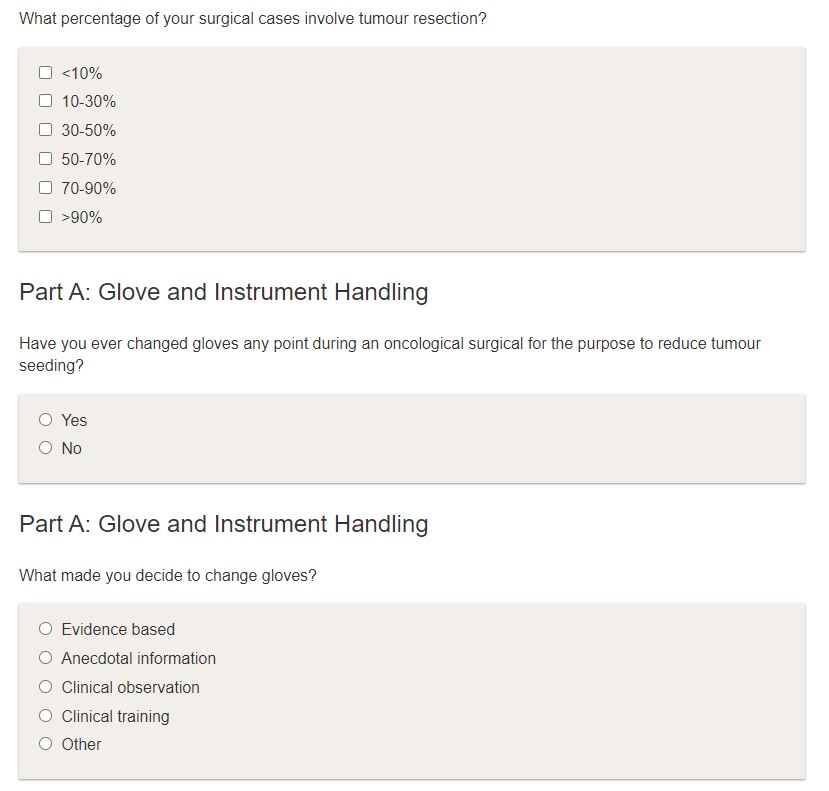
Appendix 1.


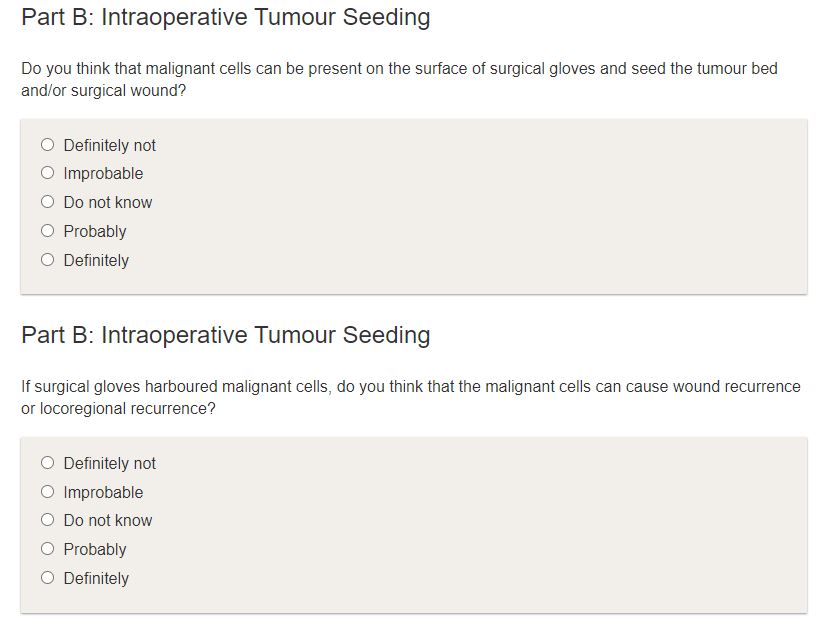

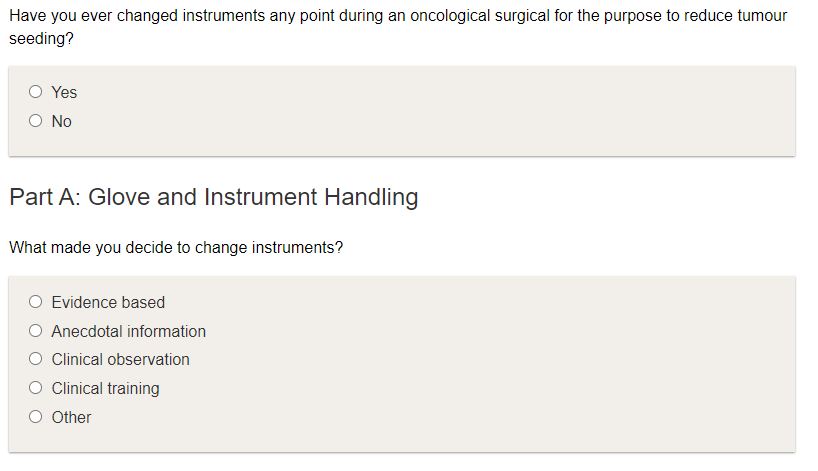
Appendix 1.


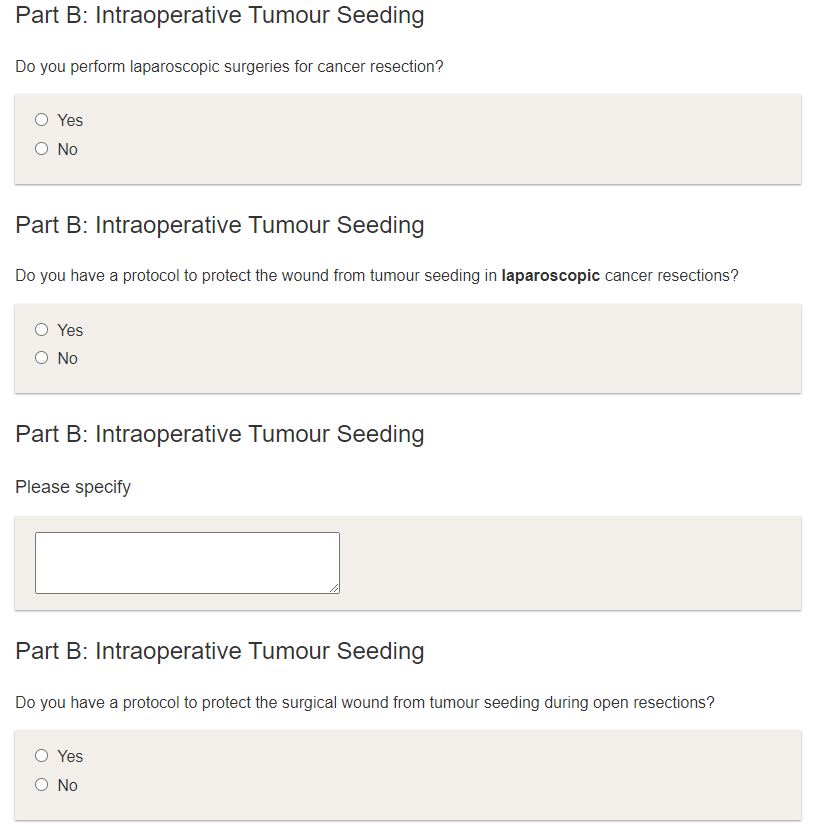
Appendix 1.


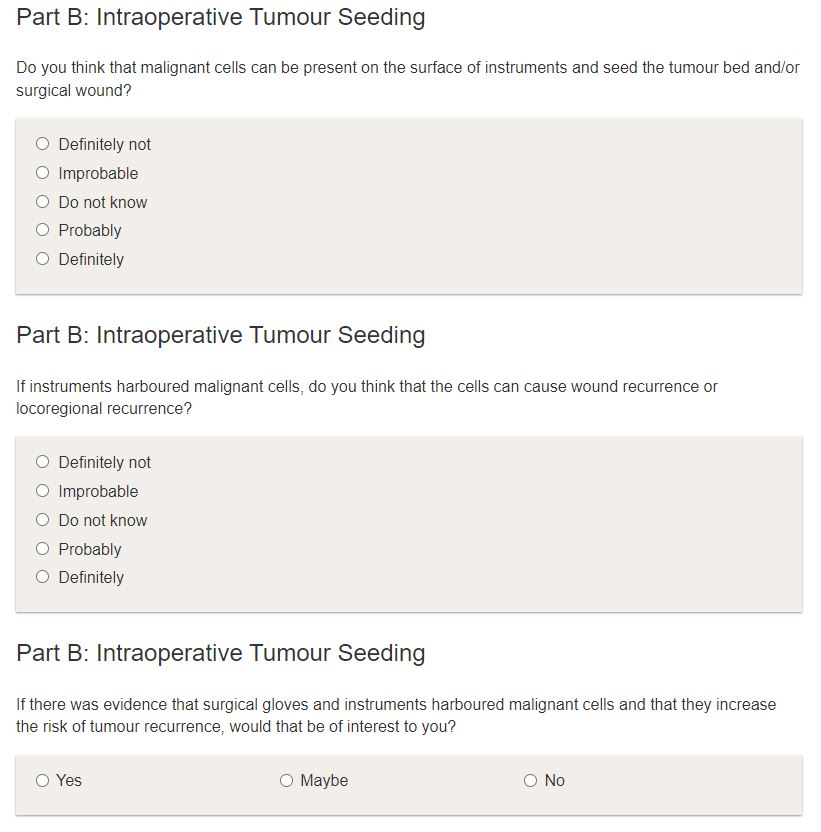
Appendix 1.

Supplement: Supplementary file 1 — Appendix S1. Questionnaire to determine veterinary surgeons' practices relating to glove and instrument handling during oncological surgeries. [file JSAP-66-477-s001.docx]
